# Viscoelastic Response of Sugar Beet Root Tissue in Quasi-Static and Impact Loading Conditions

**DOI:** 10.3390/s25123725

**Published:** 2025-06-14

**Authors:** Paweł Kołodziej, Krzysztof Gołacki, Zbigniew Stropek

**Affiliations:** Department of Mechanical Engineering and Automation, Faculty of Production Engineering, University of Life Sciences in Lublin, 20-950 Lublin, Poland; zbigniew.stropek@up.lublin.pl

**Keywords:** sugar beet root, viscoelastic properties, impact test, generalized Maxwell model

## Abstract

This paper presents the results of quasi-static tests carried out using a texturometer and of impact tests combined with stress relaxation on a stand equipped with a heavy pendulum of the hammer type. The tests were carried out using fresh roots and those stored at 20 °C for 120 h. The impact velocities *V_d_* were 0.001, 0.002, 0.01, 0.02, 0.75, and 1.25 m·s^−1^. Compiling the relaxation times *T*_1_ for *V_d_* indicated their large drops for both fresh and stored roots. The largest average values *T*_1_ were obtained in the range from 0.197 s to 0.111 s at the small velocities of deformation 0.001–0.02 m·s^−1^ and the smallest ones in the range from 0.0252 to 0.0228 s at the *V_d_* equal to 0.75 and 1.25 m·s^−1^. A decrease in *T*_2_ values was observed in the average range of 8.02–4.27 s at *V_d_* = 0.001–0.02 m·s^−1^ for fresh beets. For the velocities 0.75 m·s^−1^ and 1.25 m·s^−1^ and stored roots, the range of average values was smaller and ranged from 6.13 s to 4.54 s. The reaction forces of the *F_p_* sample reached the highest average levels from 168.2 N to 190.8 N for fresh roots and 46.5 to 56.2 N for 5-day-old roots. However, the lowest *F_p_* was recorded at speeds (0.001–0.02 ms^−1^) 57.5–62.3 N for the fresh roots and 46.5–56.2 N for the 5-day-old roots. For the velocities greater than 0.75 m·s^−1^ and 1.25 m·s^−1^, the values of reaction forces increased at the average values 168.2–190.8 N for the fresh roots and 158.2–175.4 N for 5-day-old ones.

## 1. Introduction

Quality improvement leading to greater production usability of raw beet material resulting from sugar producers’ expectations is of significant importance and requires new research methods as well as modification of those already existing, determining root susceptibility, e.g., to mechanical damage. They cause both quantitative and qualitative losses of beets during harvesting, transport, and storage [1,2,3,4,5]. The activities confining losses need investigations of various properties of roots including the following characteristics: physical (e.g., mechanical, geometrical), biological (e.g., morphological, anatomical), and chemical (e.g., tissue structure, cellular juice composition) [6,7,8,9]. Working elements of agriculture machines, e.g., beet diggers, cleaning stars, and warm and rod transporters moving during work with great velocities cause short duration force impulses (lasting milliseconds). Therefore, a great number of root defects are caused by loads of impact character [10,11,12].

Mechanical damage of beets increases the loss of sucrose during storage, which becomes “energetically” used up for filling the damaged tissue of the root with the cork suberine. This results in the drop of technological applicability of roots [13,14,15]. Resistance to mechanical loading and, associated with it, properties of beet tissues were the subject of numerous investigations using the quasi-static tests of compression, puncture, creep, or stress relaxation [16,17,18,19,20]. However, the reaction of root tissue to the impact load that takes place when the velocity of impact is above 0.25 m·s^−1^ was studied to a smaller extent. In the case of vegetables and fruits such as apples, pears, tomatoes, or avocados, even such small values of velocities and accompanying forces cause large quantitative and qualitative losses mainly due to bruises [21,22,23]. The literature reports on the results of impact of beets are fewer than in the case of quasi-static tests. For example, in the paper by Bentini et al. [24], the authors carried out the impact investigations using the rigid pendulum fastened with a beet with which they struck a steel plate with various velocities. Similar tests are discussed in the paper [25]. In the experiments, there was a cord pendulum applied for suspending roots and a high-speed photo camera to register the sequence of the beet impact against the rigid surface. The authors determined the impact energy, absorbed energy, and maximal impact force. Another kind of impact study using a laser vibrometer was carried out in the paper by Trnka et al. [26]. From the root reaction to the stroke with the impactor, the researchers stated that maximal values of impact force and displacement can be used for estimation of beet quality, e.g., depending on the surface shape, load values, or storage time. The authors found also that the maximal force increases with the impact velocity and decreases depending on the storage time. Moreover, the test results also confirm the viscoelastic properties of root tissue. They result from the dependence of forces of plant material reaction to the deformation velocity increase during the impact. However, difficulties in determining the sizes of damage areas taken into account, in which the structure reactions resulting from exceeding the values of loads in the form of blue spots do not occur, as it takes place, e.g., in the case of apples, pears, or potatoes. The sugar beet roots possess such a compact ring-shaped structure. The internal structure and the high turgor of cells favor, especially during harvesting and transport, expansion of damages that will affect the quality of root structure during storage [27,28,29]. For the description of viscoelastic properties of chosen materials in both quasi-static, e.g., creep or stress relaxation and impact investigations, there can be various phenomenological models applied (Kelvin–Voight, Burgers, Zener, and Maxwell). They are configurations in parallel and are arranged in rows as a combination of springs and dampers, making a model [30,31,32]. The estimation of the chosen set of combinations for the description of phenomena of stresses relaxation or creep is preceded by the determination of values of rigidity (*E_i_*) and suppression (*η_i_*) coefficients characterizing the model. They describe the exponential curve, which approximates the actual course of biological material reaction force against different external loads.

The aim of this study was to determine the effects of the initial deformation velocity and storage time on the parameters of the generalized Maxwell model during stress relaxation in sugar beet root samples. An attempt was made to interpret the changes in the obtained model parameters in relation to damage phenomena occurring within the tissue during and after sample loading.

## 2. Materials and Methods

**Figure 1:**
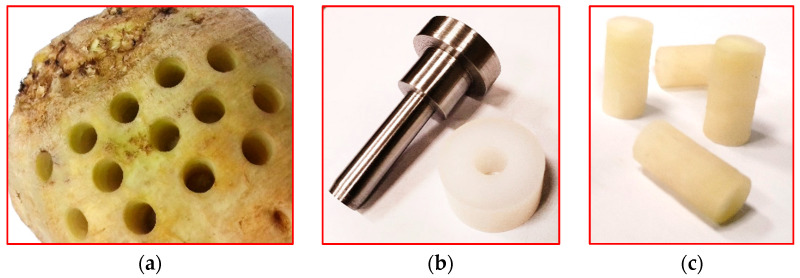
Materials used in the impact and stress relaxation tests: (**a**) a root with an area for obtaining samples; (**b**) a tubular chisel and a liner for obtaining the required length of the samples; (**c**) the samples cut out from the sugar beet.

Sugar beets of the “Zorian” variety meant for investigations were obtained from the farmstead located in the village Sieprawki (51°17′54 N 22°22′52″ E) in the Lublin region (Poland). Assuming that the results of mechanical stroke due to root impact against mobile mechanisms of gathering and transporting machines are found to the depth of ~20 mm from the beet outer surface, we took the volume round its longitudinal axis formed by this size as the space for obtaining samples (Figure 1a). The samples cut out from the roots were placed in the liner of the inner diameter, corresponding to their diameter and trimmed to obtain a required length (Figure 1b).

**Figure 2:**
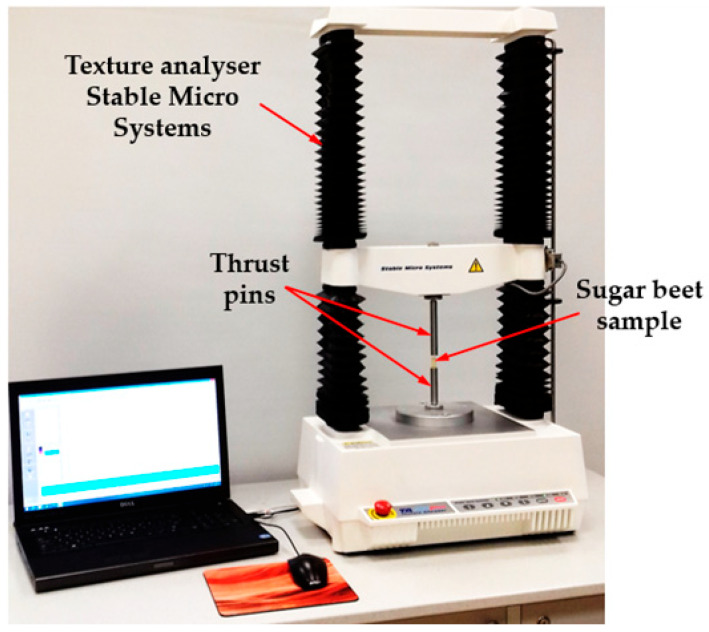
Measuring stand for the tests in quasi-static loading conditions - the texture analyzer (model TA.HD plus, Stable Micro Systems, Goldaming, UK) with a computer and a sugar beet sample between thrust pins.

The prepared samples of 11 mm diameter and 15 mm length (Figure 1c) were subjected to stresses at the deformation velocities, with *V_d_* being 0.001, 0.002, 0.01, 0.02, 0.75, and 1.25 m·s^−1^. The deformation value *d_p_* was taken as 10% of the sample height in both quasi-static and impact tests. Both fresh roots and those stored for 5 days at a temperature of 20 ± 1 °C and an air humidity of 50 ± 2% were investigated. The quasi-static tests were carried out using the texturometer Model TA.HD plus produced by the firm Stable Micro Systems, Goldaming, UK (Figure 2), equipped with the cell load for the size of 30 kg. Together with the computer and software, it made a slotted line for the registration of reaction force course in time at the sample loading.

**Figure 3:**
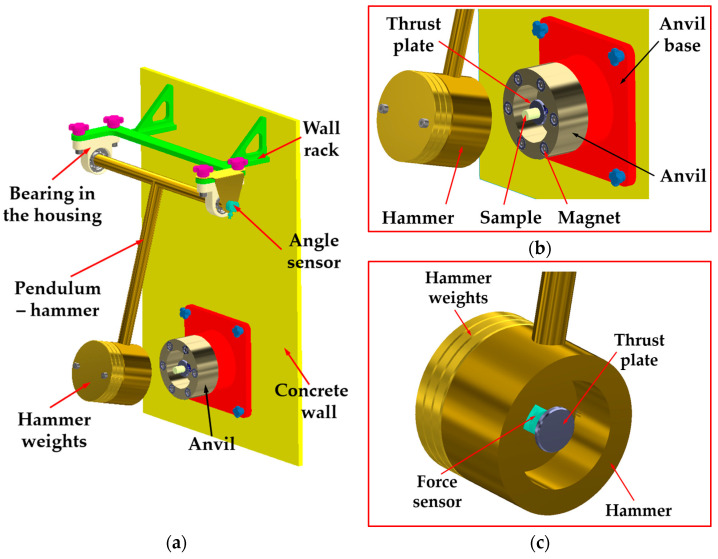
Scheme of the measuring stand for impact and stress relaxation of sugar beet root samples: (**a**) the stand view; (**b**) the anvil with the resistance surface and the test sample; (**c**) the pendulum–hammer with the force sensor.

The stand to study the sample reaction to the impact was built of a pendulum machine with three weights, with the total mass of 4500 g enabling the obtaining of kinetic energy, allowing for the sample deformation with close to constant impact velocity (Figure 3a). The arm of the pendulum of the length 940 mm enabled linear dislocation of the hammer both before and during the impact of the sample (deformation *d_p_* = 1.5 mm).

The pendulum axis was supported on the rolling bearings placed in cases connected with the rack fixed to the vertical wall. On the pendulum axis, we placed an angle sensor (WMU45SK–WObit, Pniewy, Poland), which made it possible to determine the impact velocity measuring the arm deflection from the perpendicular. Figure 3b presents the anvil of the hollow cylinder shape fixed in the holder built into a vertical concrete wall. The force of hammer impact on the sample was measured by means of the piezoelectric sensor—Endevco model 2311–100 (Meggitt Sensing Systems, Irvine, CA, USA) of a sensitivity of 24.23 mV∙N^−1^ in the measurement range of ±220 N (Figure 3c).

**Figure 4:**
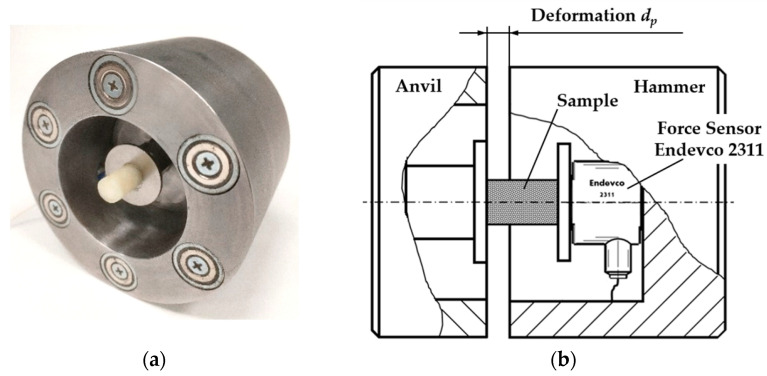
Set of impact investigations: (**a**) view of the anvil with the neodymium cylindrical magnets and the cylindrical sample fixed to the resistance plate of the sensor; (**b**) the scheme of the system: anvil–hammer with the determined distance of deformation of the sample.

The very high resonance frequency of the sensor—model 2311–100 enabled measurements of momentary, fast built-up courses of sample reaction force. The sensor was equipped with the inner hybrid conditioner of the signal in the two-conduit system, enabling transmission of the initial low impedance tension by the conduit used for supplying direct current. Signal earthing was connected to the outer encapsulation of the sensor. Being fixed between the sample and hammer, it measured the impact force precisely, not changing characteristics of the mechanical system. A recorder of the signal as a measuring path along with the force sensor and the computer worked with the sampling frequency being 2.06 kHz. In the anvil holes arranged in the circular array (Figure 4), there we placed 6 neodymium magnets (Elesa–Ganter, Stara Iwiczna, Poland) of the attraction force of 27 N each in order to stabilize the hammer position in relation to the anvil at the impact time. Cylindrical samples were attached to the vertical resistance plate of the sensor by means of technical Vaseline (Figure 4).

The compression and stress relaxation test under impact loading conditions (at velocities of 0.75 m·s^−1^ and 1.125 m·s^−1^) required the manufacture of a special impact stand described above. This stand (Figure 3), designed and built by the authors of the paper, required adaptation to beet root tests. The essence of the research was to determine the effect of the deformation velocity on the model parameters, which for an ideally viscoelastic material and the used modeling method should take the same values, regardless of the used deformation velocity. Stress relaxation in the material was studied by using a load in the form of ramp step function and measuring the decreasing reaction force of the sample over time. Other biological materials mentioned in the paper were tested in the same way. Adapting the stands to test a hard material such as sugar beet root involved, among other things, increasing the load on the hammer, installing 6 magnets to ensure a constant deformation velocity during the impact, and adjusting the size of the deformation.

The generalized five-parameter Maxwell model was used for the description of the force of sugar beet tissue reaction to loading. Taking into account the deformation velocity *v* and the diameter *d_p_* of the sample compressed along the length *l_s_*, the force course as a function of time *F*(*t*) was presented by means of the modified dependence [33]:

(1) $$F\left(t\right)=\left[\frac{S\cdot v}{l_{s}}\cdot \int_{0}^{t_{m}}\left(E_{3}+\sum_{i=1}^{2}E_{i}\cdot e^{-\frac{E_{i}}{\eta_{i}}\cdot \left(t_{m}-t\right)}\right)dt\right]\cdot e^{-\frac{E_{i}}{\eta_{i}}\cdot \left(t-t_{m}\right)}$$

where: *F*(*t*)—the force at time [N], *S*—the transverse section area of the sample [m^2^], *v*—the deformation velocity [m∙s^−1^], *l_s_*—the length of sample [m], *t_m_*—the time of deformation [s], *t*—the time from the beginning of sample deformation [s], *E*_3_—the equilibrium modulus [MPa], *E_i_*—the elasticity modulus [MPa], and *η_i_*—viscosity coefficient [MPa·s].

In the process of stress relaxation, there are two stages: the first one that is the uniaxial compression in which deformation and reaction force *F*(*t*) of the beet tissue increase to the maximal value, and the other one in which deformation becomes established and the reaction force decreases in time. Dependence (1) was used for description of the second part of investigations for the time (*t > t_m_*). It takes into account the sample relaxation during compression. Thus, the deformation velocity *v* and the deformation time *t_m_* are included in the formula. The courses of sample reaction forces obtained from the relaxation tests were approximated with dependence (2).

(2) $$F\left(t\right)=A_{e}+\sum_{i=1}^{2}A_{i}\cdot e^{-\alpha_{i}\cdot \left(t-t_{m}\right)}$$

where: *A_e_*, *A_i_ α_i_*—the parameters of the equation describing the model.

For determination of the values of parameters (*A_e_*, *A_i_*, *α_i_*) of the above equation, we applied the non-linear method of quasi-Newton minimalization in the package Statistica 13 (StatSoft, Tulsa, OK, USA).

(3) $$E_{3}=\frac{A_{e}\cdot l_{s}}{S\cdot v\cdot t_{m}}$$

(4) $$E_{i}=\frac{A_{i}\cdot l_{s}\cdot \alpha_{i}}{S\cdot v\cdot \left(1-e^{-\alpha_{i}\cdot t_{m}}\right)}$$

(5) $$\eta_{i}=\frac{A_{i}\cdot l_{s}}{S\cdot v\cdot \left(1-e^{-\alpha_{i}\cdot t_{m}}\right)}$$

The courses of force obtained during the investigations were approximated by means of scores of measuring points. The number of points and sampling time were chosen depending on the time interval and intensity of changes of the stress relaxation course. In the first interval to 0.1 s, we chose 11 points every 0.01 s, then 10 points in the interval to 1 s every 0.1 s, next 10 points in the interval to 10 s every 1 s, and the last 5 points in the interval to 35 s every 5 s. *A*_1_, *A*_2_, and *A_e_* as well as *α*_1_ and *α*_2_ were calculated from equations (3–5). Then, comparing dependences (1) and (2), we determined the values of parameters of the generalized Maxwell model (*E*_1_, *E*_2_, *E*_3_, *η*_1_, and *η*_2_).

The investigations were preceded by measuring samples mass using the scales AS 110.R2 with a measurement range of 110 g and a scale interval of 0.0001 g. After carrying out the tests, the samples were dried according to the procedure ASAE 2012 [34] in the chamber for the thermal investigations KBC–65W (WAMED, Poland). The dried samples were weighed and their moisture, that is, the water content in the damp material was calculated from the dependence:

(6) $$WSB\%=\frac{M_{k}-M_{b}}{M_{b}}\cdot 100$$

where *WSB*% is the moisture content [%], *M_k_* is the mass of root sample in grams before drying [g], and *M_b_* is the mass of the sample after drying [g].

## 3. Results

**Figure 5:**
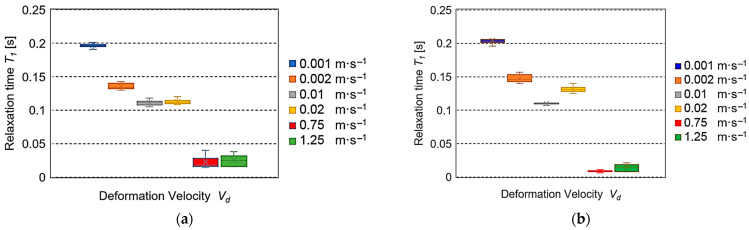
Dependence of relaxation time *T*_1_ on velocity *V_d_*: (**a**) for fresh roots, (**b**) for roots after 5-day storage.

Figure 5 presents the values of relaxation time *T*_1_. The largest average values in the range from 0.197 s to 0.111 s (at the boundary values: 0.208–0.105 s) were achieved by *T*_1_ at the small deformation velocities 0.001–0.02 m·s^−1^ and the smallest ones in the range from 0.0252 s to 0.0228 s (the boundary values: 0.04–0.016 s) at the large values 0.75 m·s^−1^ and 1.25 m·s^−1^.

**Figure 6:**
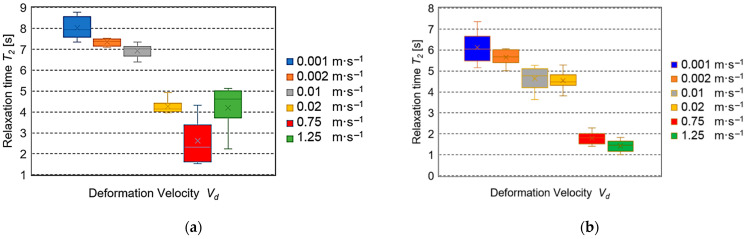
Dependence of relaxation time *T*_2_ on velocity *V_d_*: (**a**) for fresh roots, (**b**) for roots after 5-day storage.

Figure 6a presents the *T*_2_ values in the average value range from 8.02 s to 4.27 s (the boundary values: 8.77–3.96 s) at quasi-static deformation velocities *V_d_* = 0.001–0.02 m·s^−1^ for fresh beets. In the case of the velocities from 0.75 m·s^−1^ and 1.25 m·s^−1^ for the 5-day-old roots (Figure 6b), the average values *T*_2_ were from 6.13 s to 4.54 s (the boundary values: 7.35–3.63 s). However, for the greater values (0.75–1.25 m·s^−1^), the average relaxation times *T*_2_ were in the range of 1.79–1.42 s and the boundary values were in the range from 2.28 s to 1.00 s.

**Figure 7:**
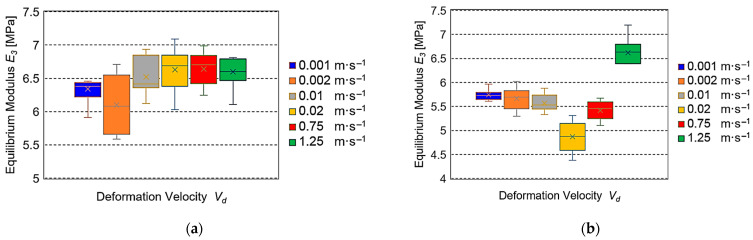
Equilibrium modulus *E_3_* on velocity *V_d_*: (**a**) for fresh roots, (**b**) for roots after 5-day storage.

Figure 7 presents the observed equilibrium values of modulus *E*_3_ depending on *V_d_*.

**Figure 8:**
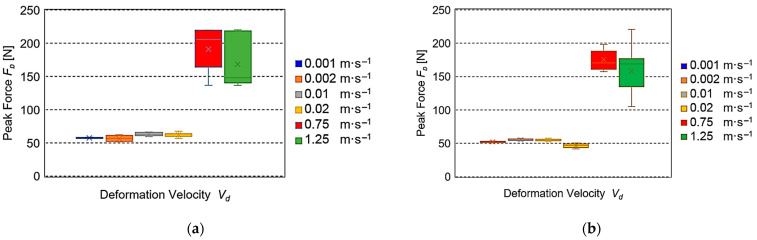
Dependence peak force *F_p_* on velocity *V_d_*: (**a**) for fresh roots, (**b**) for roots after 5-day storage.

Figure 8 presents the dependence of reaction forces of the sample *F_p_* for six deformation velocities. The smallest values were found for low velocities (0.001–0.02 m·s^−1^), being on the average from 57.5 to 62.3 N for the fresh roots and 46.5 to 56.2 N for five-day-old ones. For the higher velocities of 0.75 m·s^−1^ and 1.25 m·s^−1^, *F_p_* achieved the average values from 168.2 N to 190.8 N for the fresh roots and 158.2 to 175.4 N for 5-day-old ones.

**Figure 9:**
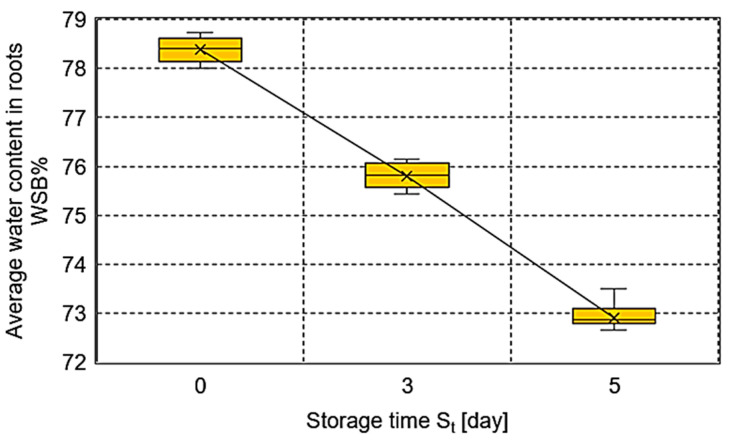
Average water content, *WSB*%, in the beet root samples depending on the storage day.

The water content in ten samples cut out from the roots, which was determined based on Equation (6), was averaged and is presented in relation to the storage day in Figure 9. The average moisture content (*WSB*%) in the exterior root layer of the root changed with the storage time and changed from the range 78.743–78.008% to the range 73.493–72.180% under the conditions of the 5-day experiment. Moreover, a statistical dependence between the moisture content and storage time *S_t_* with a correlation coefficient of *R*^2^ = 0.97 was found.

## 4. Discussion

The plant material of the beet is a complex structure formed by single cells of the root surrounded by the intercellular gases and fluids, which when subjected to mechanical loads, show a tendency towards relaxation, resulting in the disappearance of the forces in time. The generalized five-element Maxwell model taken for description of sugar beet tissue behavior being loaded is presented as a system of suitably combined elastic (three springs) and sticky (two dampers) elements. The three branches of the model set up in parallel include two made from the series combinations of spring–damper and one includes only a spring. They are characterized by time constants in the form of the quotients called the relaxation times *T*_1_ = *η*_1_*·E*_1_^−1^ and *T_2_* = *η*_2_*·E*_2_^−1^, as well as the *E*_3_—equilibrium modulus constant [35,36]. Their values indicate intensity of liquid and gas transport in the root intercellular structure between the areas of different internal pressures due to the applied load and stabilization of the stress (force) after time.

The results of the experiments conducted for the assumed values of deformation velocity *V_d_* are presented in Figure 5, Figure 6, Figure 7 and Figure 8. The cellular flow of gases with the load is the fastest. Therefore, it is expressed by a time constant of the seconds fraction value—relaxation time *T*_1_ (*η*_1_*·E*_1_^−1^). The specification of relaxation times *T*_1_ for different deformation velocities *V_d_* presented in Figure 5a,b indicated their large drops observed for both fresh and stored roots. This trend is consistent with the results of investigations presented in the papers concerning apples, pears, and potatoes [37,38], as well as for biodegradable films [39]. It should be emphasized that the relaxation times *T_1_* obtained in the range from 0.001 m·s^−1^ to 0.02 ms^−1^ were 10–30 times higher than those registered for *V_d_* = 0.75 m·s^−1^ and 1.25 m·s^−1^. The motion of intercellular fluids in the Maxwell model can present the relaxation time *T*_2_ (*η*_2_*·E*_2_^−1^). Their flow is slower than that of gases, so the obtained values *T*_2_ are expressed in seconds. Figure 6a presents a huge drop of *T*_2_ in the range of average values at quasi-static deformation velocities *V_d_* = 0.001–0.02 m·s^−1^ for fresh beets. In the case of velocities from 0.75 m·s^−1^ and 1.25 ms^−1^ for the stored roots (Figure 6b), the drop was also observed, but its range of average values was smaller. Similar behavior of loaded beet tissue was observed at greater deformation velocities *V_d_*, being 0.75 m·s^−1^ and 1.25 m·s^−1^ for the 5-day-old roots. It should be stressed that the average relaxation times *T*_2_ were over 3.5 times smaller than those registered for the quasi-static velocities. The other parameter describing the courses of viscoelastic material stress relaxation is the equilibrium modulus *E*_3_, which in the generalized Maxwell model can represent an element of a very long time constant (stable) that is the value after stabilization of stress (force). In this paper, the values *E*_3_, depending on the deformation velocity, are presented in Figure 7. For fresh roots, insignificant changes *E*_3_ were found for the quasi-static deformation velocities in the range 0.001–0.02 m·s^−1^. However, in the velocity range 0.75 m·s^−1^ and 1.25 m·s^−1^, the values *E*_3_ were comparable (Figure 7a). They differed significantly from the results obtained, e.g., for apples, for which at large deformation velocities, the values of the equilibrium modulus *E*_3_ were much smaller than those observed for the quasi-static deformation velocities [38,40]. However, in the case of sugar beets, it can be suggested that this is the effect of a small content of gases in the tissue structure. This results in small deformation formed during annihilation of the spaces filed with gases between the beet cells, which can arise at the loads with the deformation velocities in the range m·s^−1^. Moreover, it should be stated that at the assumed values for the experiment deformation value and deformation velocity, a not very large destruction of sample structure due to the action of impact load can take place. In the case of maximal reaction forces of the sample *F_p_*, the smallest values were found for low deformation velocities for both fresh and five-day-old roots. For the higher velocities, a 3–4 times increase in *F_p_* took place for fresh beets, which was observed also for 5-day-old roots. This trend was also observed for 5-day-old roots. This increase was the result of over 60 times larger impact velocity. The differences in the *F_p_* values took place after storage and indicated a decrease in the average reaction force in the range 9–19% for the velocity 0.001–0.02 m·s^−1^ and 6–8% for *V_d_* = 0.75 m·s^−1^ and 1.25 m·s^−1^. The decrease in average values of reaction forces observed for all velocities for the 5-day-old roots was due to a decrease in water amount in the beet external layers (softening of the tissue) after the storage.

In the world literature, we do not find studies of visco-elastic parameters of samples or whole roots under both quasi-static and impact mechanical loading conditions. However, the magnitudes characterizing visco-elastic rheological models are useful for studies of the bruise sensitivity of fruits and vegetables, as well as for describing the behavior of tissue during long-term load. This is supported by the results of experiments conducted by Zhang et al. [41] on Chinese cabbage, Wang et al. [42] on Korla Pearl, and in studies of apples [43], as well as in the work on microstructure studies of tomato under loading [44].

A novelty of this work was the setting of the deformation constant in the form of a ramp step function, which made it possible to compare the parameters of the Maxwell model over a wide range of deformation velocities. In the papers of other authors, where stands equipped with pendulums of different types were used, only the impact velocity was controlled, without influencing the amount of deformation. These were impact tests conducted, for example, on peaches [45], kiwifruit [46], and pomegranate [47] but also litchi [48]. The results of this research increase the knowledge of the mechanical characteristics of different biological materials. However, their different tissue structures and different proportions of air spaces and water content limit comparisons in terms of trends and overall similarities. Based on the results of the study presented in this paper, it can be concluded that the nature of the changes in relaxation times (*T*_1_, *T*_2_) had the same downward trend, but their values were different for apples [38]. The phenomenon can be explained by a different percentage of air spaces as well as structural and moisture differences in the tissue.

It should also be noted that the damage of plant tissue can occur as a result of exceeding the permissible value of deformation as well as the speed of deformation. This can occur in the case of viscoelastic materials, which include most fruit and vegetables as well as sugar beet as an industrial plant.

There is a loss of water during the storage of fruit and vegetables, the intensity of which is largely related to ambient conditions. In particular, it is the temperature and humidity and, in addition, the respiration surface. In fruit, there is then a softening of the tissue and a decrease in the bonding strength between cells. This results in a disruption of physiological functions due to an increase in cell membrane permeability, which becomes deformed. Progressive structural changes during storage accelerate biochemical reactions in the fruit. In the case of sugar beet, the root “softens” from the outside in, which is why samples taken from the superficial layers showed a decrease in the sample impact reaction forces for 5-day-old roots compared to fresh roots. In the present study, we determined the water content (*WSB*%) of the samples during the entire test. The storage parameters: temperature 20 ± 1 °C and the air humidity 50 ± 2% resulted in a decrease in the water content of the samples ranging from 78.7 to 72.2% under the conditions of the 5-day experiment. This is comparable to the results presented in Al-Jbawi at al. [49]. The authors obtained decreases in average root water content in the range of 75.24–60.02% during a 10-day storage period with a post-harvest temperature range of 20.10–37.50 °C.

From a practical point of view, it is important to determine the critical drop height at a certain root weight, but there are many other aspects that require in-depth knowledge of the beet’s mechanical characteristics, such as the resistance of the roots to the ploughing elements in the soil, the transport mechanisms of the harvester, or the long-term loading of the beet in the heap during storage.

One of the objectives of the study was to identify viscoelastic characteristics of sugar beet roots over a wide range of deformation velocities. The results showed significant differences in the values of the model parameters as well as the maximum reaction force of the samples under quasi-static and impact loading conditions. It should be believed that the applied test method that defects such differences is worth implementing to other varieties of beets in the future as well.

## 5. Conclusions

1. The determined relaxation times *T*_1_ showed the largest values 0.197 s for the deformation velocity 0.001 m·s^−1^ and decreased significantly with the increasing *V_d_* to the 0.0228 s for the velocity 1.25 m·s^−1^. Here, the decrease was observed for the quasi-static velocities and for the impact ones. Such a response of the beet tissue to the load was observed for both fresh and stored roots.
2. The highest values of the relaxation time *T*_2_ were found for fresh roots, and at the quasi-static deformation velocities, they were 8.02 s for 0.001 m·s^−1^, but the smallest ones were at the impact velocities. For 5-day-old roots, the average relaxation times *T*_2_ were in the range 1.79–1.42 s, being 3.5 times smaller than those registered for the quasi-static velocities.
3. The highest average values of reaction forces *F_p_* were found for the impact deformation velocities. For the quasi-static *V_d_* values, the average *F_p_* were 3–4 times smaller than under impact loading conditions. It can also be concluded that the observed increase in the force response value within increasing deformation velocity confirms the viscoelastic nature of the investigated material.
4. The values of Maxwell model parameters can be used to assess the susceptibility of sugar beet root tissue to initial damage under various loading conditions.

## Data Availability

Data are contained within the article.
